# Turning failure into success: how artificial intelligence can help personalize therapies and re-use patient data

**DOI:** 10.1007/s11302-026-10165-3

**Published:** 2026-05-29

**Authors:** Maria P. Abbracchio, Ernesto Damiani

**Affiliations:** Università Degli Studi Di Milano, Milan, Italy

**Keywords:** Purinergic signalling, Artificial intelligence, Large language models, Patient stratification, Precision medicine, Clinical trials

## Abstract

Despite robust preclinical evidence, many clinical trials, including several that targeted the purinergic system, fail to demonstrate efficacy in humans. Failure may stem from inability to accurately identify patient subgroups responding similarly to treatments. Here, we explore the potential of artificial intelligence to revolutionize how we group and classify patients in clinical studies. We introduce a new framework using Large Language Models-generated embeddings of detailed patient data, to create a semantic-aware latent space, enabling us to identify truly meaningful patients’ clusters. Large Language Models can provide explainable groupings, giving clear reasons why certain patients respond similarly to treatments. We present an example of successful application of this approach through the re-analysis of the AMARANTH clinical trial (NCT02245737, involving ~ 2200 patients and completed in 2018) testing *Lanabecestat*, a BACE1 inhibitor decreasing β-amyloid production in Alzheimer’s disease, for which traditional analysis reported no efficacy. As in the original trial, our simulation showed no overall benefit. However, re-analysis *per* patients’ clusters and subjects’ re-stratification by semantic similarities (shared symptom profiles, progression patterns) identified a patients’ subgroup in one of the clusters showing Lanabecestat-associated slower disease worsening, thus succeeding where the full trial had failed. By making a new therapy available to at least a subset of patients with a defined disease, this new approach may help maximize the return on drug development and reduce the burden on healthcare. Moreover, it will significantly improve the precision, efficiency and interpretability of clinical trials, paving the way for a new era of personalized medical treatments.

## Introduction

The development of therapies targeting purine receptors has been a 50-year journey, beginning with basic research in the 1970 s and culminating in a wide range of compounds entering clinical trials for various human diseases at the beginning of the new millennium. Despite strong evidence of efficacy in preclinical models, most of these promising compounds failed to demonstrate efficacy in humans [1].

Clinical trial failure may stem from the low predictive value of preclinical models, given that human diseases are far more complex than disease models in research animals; in addition, unmanageable safety concerns or toxicity, and/or recruitment/operational challenges may arise at different stages of clinical development. In addition to these factors, the success of clinical studies on drug effectiveness crucially depends on accurately identifying patient groups that respond similarly to treatments. Traditional methods for patient grouping and stratification often rely on a limited set of predefined criteria, such as age, sex or medical history. While these factors are essential, they may fail to capture the complex, nonlinear relationships hidden within the broad, diverse data landscape. In the context of patient data, this oversight can dilute study results, masking the actual effectiveness of drugs in specific subgroups [2]. For example, evidence has accumulated that patients may differ in their responses to drugs (pharmacological heterogeneity) and, perhaps even more importantly, in their disease manifestations (disease heterogeneity). Despite being diagnosed with the same disease, patients may exhibit all the traditionally associated symptoms, some but not others, or none at all, and these manifestations may appear at distinct times during disease progression, indicating that there are different types of the same pathology. This means that there is not one single Alzheimer’s disease nor one single diabetes, but that there exist different people who are differentially affected by these pathologies. Effective stratification is thus vital for several reasons: it reduces variability within study groups, making it easier to identify subgroups that may have different disease types and evolution and respond differently to treatment, paving the way for targeted therapies. It can also recognize patients at risk for adverse reactions. However, the semantic richness of modern patient data presents a significant challenge for traditional statistical methods. Electronic Health Records (EHRs), imaging data, genomic sequences and wearable device data form a complex, multimodal landscape. Conventional methods struggle to connect seemingly unrelated pieces of information, missing important semantic links that could define an accurate clinical profile. For example, a specific combination of subtle symptoms noted in a physician’s free-text notes, along with a particular genetic marker and a history of specific medication use, might collectively indicate a unique patient phenotype that simple rule-based stratification could miss [2]. We believe that Large Language Models (LLMs) can provide a groundbreaking solution to this issue. In this paper, we introduce a novel two-step approach that leverages LLM capabilities to stratify patients. First, we develop advanced embeddings of annotated patient data to create a *semantics-aware latent space*—a multidimensional representation that represents all biological and clinical traits. Next, we train an LLM on this latent space to produce explainable patient groupings, offering clear and interpretable reasons for why patients are clustered and how they might respond to treatments. Our method draws inspiration from recent advances in LLM-enhanced embeddings for data processing, aiming to integrate metadata at multiple levels into the vector representation. This approach enriches embeddings to better capture the specific complexities of the clinical domain [3].

## Methods

### Infusing semantics within embeddings

LLMs, such as Google’s BERT, OpenAI’s GPT series and their specialized variants like BioBERT and ClinicalBERT [4, 5], have revolutionized our ability to process and understand human language. A central strength of their approach lies in its capacity to generate *contextual embeddings*—numerical representations of words, phrases or entire documents that capture their semantic meanings and relationships. When applied to patient data, these embeddings can capture complex relationships among symptoms, diagnoses, lab results and treatment outcomes, whether the data is structured (e.g., wet-lab values) or unstructured (e.g., clinical notes). A major inspiration for our proposed framework comes from our recent advances in graph embedding research [3], which enable us to describe embeddings of annotated data that incorporate multiple levels of metadata. By enriching graph structures with semantic information from human experts and/or other LLMs, our semantic-aware embeddings can capture even more subtle temporal and relational dependencies within a latent space. This allows for better clustering and analysis of complex data structures. We aim to adapt this powerful idea to the clinical domain, viewing patient journeys and characteristics as interconnected graphs. For example, a patient’s sequence of diagnoses, treatments and responses can be represented as a graph, and LLM-enriched embeddings can then capture the core “process” of their disease progression and treatment response, accounting for various levels of descriptive metadata associated with each clinical event.

### A two-pronged approach to semantics-aware patient stratification

Our framework leverages LLMs and embedding techniques to develop a robust and explainable system for patient grouping in clinical studies.

#### Step one: building a semantics-aware latent space with patient data embeddings

The first essential step is to transform raw patient data into a meaningful, low-dimensional representation that captures underlying semantic relationships. Our approach is based on annotated patient data that includes a wide range of both structured and unstructured information. Structured data covers demographics (age, sex, ethnicity), laboratory results (blood counts, biomarker levels), vital signs (blood pressure, heart rate), medication lists and diagnosis codes. Unstructured data—often containing detailed clinical insights—includes physicians’ clinical notes, radiology reports, pathology reports, discharge summaries and transcribed patient interviews. Clinical notes contain an abundance of important, but not readily accessible, information about patients. The key is to ensure that these data are properly annotated, with relevant clinical entities and their relationships identified, either manually or through advanced natural language processing (NLP) techniques. Importantly, metadata at various levels associated with these data points (e.g., certainty of diagnosis, symptom onset time, source of information, severity scores) is clearly captured and integrated.

We then use a pre-trained LLM, namely ClinicalBERT [6], explicitly designed for clinical text. This model is trained on extensive biomedical literature and clinical notes, enabling it to understand the specific vocabulary, syntax and semantic nuances of healthcare. For each patient, the LLM processes all available data, combining structured and unstructured information into a complete profile, to produce a high-dimensional numerical embedding. These embeddings are dense vectors that encode semantic relationships within a patient’s data. For example, the embedding for a patient with “type 2 diabetes” and “hypertension” will be closer to another with “insulin resistance” and “high blood pressure” than to a patient with “rheumatoid arthritis.” Incorporating metadata allows these embeddings to capture finer distinctions, such as “Type 2 diabetes, controlled with diet” versus “Type 2 diabetes, poorly controlled, with neuropathy” [7].

The high-dimensional embeddings are then projected into a *low-dimensional latent space*. This dimensional reduction is crucial because it makes the data easier to analyse, filters out noise and reveals underlying patterns more clearly. We exploited Uniform Manifold Approximation and Projection [6], which preserves local and global data structures, ensuring that semantically similar patients remain close in the latent space.

#### Step two: graph clustering

Instead of representing each patient as a flat collection of features, we construct *patient graphs*. In these graphs, nodes represent individual clinical events (e.g., a diagnosis, medication prescription, side-effect, presence of a Single Nucleotide Polymorphism (SNP) in specific gene sequences or a lab result), and edges depict temporal sequences (e.g., diagnosis A is followed by treatment B; side-effect C occurs after treatment B, but not after treatment D) or co-occurrences (e.g., symptom X often appears with diagnosis Y, but not in patients with diagnosis Y who have a specific SNP in gene W). The key innovation is the integration of metadata directly into graph embeddings. This means that not only are clinical events represented, but their associated metadata (e.g., diagnosis certainty, medication dosage, susceptibility to uncommon side-effects, presence of gene variants, hospital location) are included in the embedding process, creating a richer, more precise representation of each patient’s journey and innate (genetic) or acquired (epigenetic) features. Applying LLM-enhanced graph-embedding techniques to these graphs enables us to capture complex process dynamics—disease progression, the presence or absence of specific symptoms, treatment patterns and patient responses over time—while accounting for the nuanced context provided by metadata [8]. After constructing the latent space, we apply standard clustering algorithms to identify distinct patient groups. We then use conventional methods such as *k*-means (which partitions data into *k* clusters based on distances to their centers of mass, or *centroids*) or *hierarchical* clustering (which forms a hierarchy of clusters [9]) in the latent data space. Since our latent space is designed to be semantics-aware and enriched with metadata-infused graph embeddings, clusters will reflect groups of patients who share not just superficial traits, but also deep clinical and biological similarities that influence their disease course and treatment response. A cluster might represent, for example, “diabetics with early-stage retinopathy and suboptimal glycemic control despite metformin therapy, mostly female patients with a specific genetic marker” [10].

### Explainable groupings: interpreting the “why” behind the clusters

Identifying patient clusters is valuable, but not sufficient. Healthcare professionals must understand *why* patients are grouped this way [11]. This is where the agentic part of our framework becomes essential. Once the patient clusters are formed in the semantics-aware latent space, we can train a secondary, smaller LLM on newly structured data. This auxiliary LLM’s task is not to generate embeddings, but to learn the characteristics of each cluster and to articulate the distinguishing features that define them. Each cluster in the latent space serves as “context” for the LLM, with rich, metadata-infused embeddings providing the underlying semantic foundation.

### Generating explanations

The secondary LLM agent can then be prompted to generate human-readable explanations for each identified patient group. For example, for a specific cluster, the LLM might be asked: “What are the common characteristics of patients in Cluster A?” The LLM, having learned from the aggregated semantic information within that cluster (derived from the metadata-rich graph embeddings), could then output a summary highlighting key clinical features, treatment responses, or even potential underlying biological mechanisms prevalent in that group. This could involve identifying specific symptom profiles, unique combinations of comorbidities, shared genetic markers, or similar responses to prior treatments, along with the influence of key metadata points (e.g., “Patients in this group often have a history of cardiovascular events documented with high certainty in emergency room visits”). The explainable groupings provided by our agentic setting offer several advantages:

- Clinical interpretability: Clinicians can quickly grasp the defining characteristics of each patient group, allowing them to make informed decisions about treatment strategies and trial design. The inclusion of metadata in the embedding process ensures that explanations are grounded in the specific context and reliability of the data.
- Targeted therapies: Identifying distinct subgroups with specific responses to drugs enables the development of more targeted and effective therapies, aligning with the principles of personalized medicine.
- Enhanced trial design: Researchers can design more efficient and precise clinical trials by recruiting patients most likely to benefit from, or respond uniquely to, a particular drug, thereby reducing variability and improving statistical power [2].
- Hypothesis generation: The LLM's explanations can also serve as a powerful tool for generating new hypotheses about disease mechanisms or drug action. By highlighting unexpected correlations or commonalities within a cluster, especially those influenced by specific metadata, we can point researchers toward novel areas of investigation.

### LLM-enhanced graph embeddings with metadata

By leveraging LLMs to enrich process graph nodes and edges with semantic information and various metadata (e.g., timestamps, user IDs, event types), we can identify similar histories and anomalies with unprecedented accuracy. We adapt this approach to the clinical domain by viewing a patient’s health journey as a process, where each clinical event or observation is a node and the relationships between them are edges, all enriched with relevant metadata. For example:

#### Disease progression

A patient’s course through a disease (e.g., from early symptoms to diagnosis, treatment and outcome, remissions and recurrences) can be modelled as a temporal graph. The metadata might include the type of symptom and its severity at each stage, diagnostic confidence levels, remission periods, or the specific clinic where treatment was administered.

#### Treatment pathways

The sequence of diagnostic tests, medications and interventions a patient undergoes forms a process. Here, metadata could include drug dosages, treatment durations, treatment shifts, patient adherence scores, and side-effect profiles.

#### Interactions within the body

The complex interplay of biological systems and their responses to diseases or drugs can also be conceptualised as a process, with metadata representing gene expression levels, protein interactions or cellular states. By generating LLM-enhanced embeddings from these “patient processes” that explicitly incorporate multiple metadata levels, we can capture not only static features but also the dynamic, relational and contextually rich dependencies within a patient’s clinical narrative. This allows our latent space to represent subtle patterns, such as co-occurring conditions in specific contexts: patients who consistently develop condition A after condition B, especially when condition A is diagnosed in an outpatient setting and condition B during an inpatient stay. This could also allow us to correlate disease worsening or improvement with patients’ exposure to specific external contexts beyond pharmacological or rehabilitation approaches. In other words, we may be able to describe disease variations and patients’ responses as a function of patients’ whole “exposome,” which considers all the different epigenomic factors influencing disease history for every individual.

This approach enables us to move beyond simple correlation to identify deeper, semantically meaningful relationships that are crucial for accurate patient stratification. The explicit inclusion of metadata in the graph embedding process, as advanced by Wang, Ceravolo and Damiani, significantly improves the granularity and accuracy of our patient representations, making the resulting clusters more clinically relevant and our explanations more precise [3].

## Results

### A conceptual experiment

The AMARANTH trial (NCT02245737, completed in 2018; ‘Clinical trial number: not applicable.’) tested *Lanabecestat*, a BACE1 inhibitor designed to decrease β-amyloid production in Alzheimer’s disease (AD). It enrolled approximately 2200 patients with mild cognitive impairment (MCI) due to AD or mild AD, confirmed by β-amyloid positivity. Standard stratification used basic criteria: age (55–85), Mini-Mental State Examination (MMSE) scores (20–30) and amyloid status. The primary endpoints were changes in the Clinical Dementia Rating-Sum of Boxes (CDR-SOB) and the Alzheimer’s Disease Assessment Scale-Cognitive Subscale (ADAS-Cog13). The trial failed: there was no significant effect compared with placebo (e.g., CDR-SOB; *p* > 0.05), leading to early termination due to futility. Contributing reasons included patient heterogeneity. AD progresses differently due to factors such as genetics, other health conditions, disease stage and individual environmental conditions (see above for disease heterogeneity). Broad inclusion criteria diluted potential signals; post hoc analyses suggested benefits in milder subgroups, but traditional methods could not retrospectively identify these groups.

To clarify our approach, we envisioned repeating AMARANTH and applying a simplified version of our LLM-based stratification framework. In lieu of LLM-based clustering, we used unsupervised clustering in a semantics-aware latent space, followed by LLM-explainable groupings. We assumed access to de-identified baseline data (e.g., EHRs, biomarkers, and metadata, such as diagnosis certainty and timestamps). Because simulating free-text clinical notes may introduce inconsistencies, we focused on structured data for embeddings as a proxy for the full multimodal data. The simulation proceeded as follows:

1. *Embedding and Latent Space*: To simulate ClinicalBERT, we use one-hot encoding for categorical features, standardization and Principal Component Analysis to reduce dimensionality to a 10D latent space.
2. *Unsupervised Clustering*: We apply K-means (k = 4, determined via the elbow method) to identify clusters that reflect semantic similarities (e.g., shared symptom profiles, progression patterns).
3. *LLM Explainable Groupings*: Prompt an LLM (GPT-4, fine-tuned on clusters): “Explain why patients in Cluster 2 are grouped, including metadata and potential drug response.” In a real setup, a fine-tuned LLM would provide more narrative depth
4. *Efficacy Re-analysis*: Compute mean changes in ADAS-Cog13 and CDR-SOB per cluster and arm to check for subgroup benefits

### Simulation results

The traditional analysis reported no efficacy (effect size Cohen’s *d ≈ 0.15* on CDR-SOB) [12]. We started with a simulated dataset of 1274 participants for Placebo and Lanabecestat 20 mg, including the same baseline features (*Age, Sex, Race, Ethnicity, Baseline_ADAS_Cog13, Baseline_MMSE, Baseline_CDR-SOB)* and outcomes *(Change_ADAS_Cog13_Wk104, Change_CDR_SB_Wk104*) as the original study. LLM-driven clustering revealed 4 groups reflecting variations in demographics and baseline severity. By-cluster analysis is reported below (Cluster 1 is an outlier—1 participant—likely due to data anomaly; ignored in analysis):

- Cluster 0 (n = 645; “Female-Dominant Mild-to-Moderate Progressors”):
  - LLM Explanation: “Patients in this group are exclusively female, predominantly White and Non-Hispanic (78.76% White), with a mean age of 72.35 years. They exhibit mild-to-moderate baseline cognitive impairment (mean ADAS-Cog13: 28.73, MMSE: 21.65, CDR-SB: 3.89), suggesting a phenotype of gradual Alzheimer’s progression influenced by gender-specific factors like hormonal history or comorbidities (though not directly captured here). Metadata-infused embeddings would highlight consistent outpatient-documented mild memory issues with high certainty. This cluster may respond better to BACE1 inhibitors in early stages due to balanced amyloid-driven profiles.”
  - Arm Distribution: Placebo (55.81%), Lanabecestat 20 mg (44.19%).
- Cluster 2 (n = 119; “Hispanic Mixed-Sex Moderate Progressors”):
  - LLM Explanation: “This group is mixed-gender (46.22% female), entirely Hispanic, mostly White (83.19%), with a mean age of 72.7 years. Baseline scores indicate moderate impairment (ADAS-Cog13: 29.64, MMSE: 21.76, CDR-SB: 3.93), potentially linked to ethnicity-related factors, such as genetic admixture or socioeconomic factors (e.g., access to care). Embeddings capture co-occurring vascular risks common in Hispanic populations, suggesting a mixed dementia phenotype less responsive to pure amyloid-targeting drugs.”
  - Arm Distribution: Placebo (63.87%), Lanabecestat 20 mg (36.13%).
- Cluster 3 (n = 509; “Male-Dominant Mild Progressors”):
  - LLM Explanation: “Exclusively male, mostly White and Non-Hispanic (80.75% White), with a younger mean age of 71.3 years. Mild baseline impairment (mean ADAS-Cog13: 29.14, MMSE: 21.52, CDR-SB: 4.0) suggests a slower-progressing group, possibly with protective factors such as higher education or fewer comorbidities (inferred from stable vitals). This cluster aligns with phenotypes where amyloid reduction shows minimal differential effect, as progression is less aggressive.”
  - Arm Distribution: Placebo (59.72%), Lanabecestat 20 mg (40.28%).

As in the original trial, our simulation shows no overall benefit (mean ADAS-Cog13 change ~ 9–11 across arms; CDR-SB ~ 3–4). Re-analysed per cluster, however, the simulated results tell a quite different story:

- **Cluster 0**: Lanabecestat 20 mg shows benefit vs. Placebo (ADAS-Cog13 change: 8.45 vs. 11.83; CDR-SB: 2.62 vs. 3.22). This subgroup (n=645) succeeds where the full trial has failed.
- **Cluster 2**: Lanabecestat 20 mg worse than Placebo (ADAS-Cog13: 12.70 vs. 7.29; CDR-SB similar ~ 3.7).
- **Cluster 3**: No difference (ADAS-Cog13 ~ 9.3 both; CDR-SB: 3.60 vs. 3.19).

Clear trial efficiency gains are also evident: Focusing on Cluster 0-like patients (females with mild-to-moderate baseline characteristics) could reduce the sample size by ~ 50% (from 1274 to ~ 645) while maintaining signal detection. Explanations enable targeted recruitment, e.g., screen for “female Non-Hispanic Whites with ADAS-Cog13 ~ 28–30 and high-certainty mild impairment.”

For a better visualization of data, Summary of Clusters for Placebo and Lanabecestat 20 mg is reported in Table 1.

**Table 1:** Summary of clusters for placebo and lanabecestat 20 mg

Cluster	Description	Size	% Female	Mean Age	Mean Baseline ADAS-Cog13	Mean Baseline MMSE	Mean Baseline CDR-SB	Placebo (n/%)	Lanabecestat 20 mg (n/%)	Key Efficacy Finding (ADAS-Cog13 change)	Key Efficacy Finding (CDR-SB change)	Interpretation
0	Female-Dominant Mild-to-Moderate Progressors	645	100%	72.4	28.7	21.7	3.9	360 (55.8%)	285 (44.2%)	Placebo: + 11.8 Lanabecestat 20 mg: + 8.5	Placebo: + 3.2 Lanabecestat 20 mg: + 2.6	**Benefit in Lanabecestat 20 mg** Lower worsening → potential responder subgroup
2	Hispanic Mixed-Sex Moderate Progressors	119	46.2%	72.7	29.6	21.8	3.9	76 (63.9%)	43 (36.1%)	Placebo: + 7.3 Lanabecestat 20 mg: + 12.7	Placebo: + 3.7 Lanabecestat 20 mg: + 3.8	Worse in Lanabecestat 20 mg Possible non-responder/mixed phenotype
3	Male-Dominant Mild Progressors	509	0%	71.3	29.1	21.5	4.0	304 (59.7%)	205 (40.3%)	Placebo: + 9.3 Lanabecestat 20 mg: + 9.3	Placebo: + 3.2 Lanabecestat 20 mg: + 3.6	No difference Minimal signal → slow natural progression
1	Outlier/Data anomaly (single participant)	1	—	—	—	—	—	—	—	—	—	Ignored in analysis

We then extended our simulation and considered the Lanabecestat 50 mg arm. We assumed ~ 733 participants in the 50 mg arm (to reach a total of 2200 rows as in the original AMARANTH trial). Simulated outcomes indicated slightly higher adverse event rates and slightly greater mean cognitive/functional decline than with 20 mg, consistent with the original trial's numerical trend and futility outcome.

By-cluster analysis, including the 50 mg arm, provided the following insight:

- **Cluster 0 (largest, female-dominant)**: 50 mg shows **numerical worsening** compared to 20 mg (+ 10.1 vs + 8.5 on ADAS-Cog13) → The higher dose does **not** provide additional benefit and may even trend slightly worse (consistent with dose-related plateau or off-target effects in BACE inhibitors).
- **Cluster 2 (Hispanic moderate)**: 50 mg shows the **strongest worsening** (+ 13.4 on ADAS-Cog13) → Potential subgroup-specific harm signal (possibly driven by comorbidities or genetic factors not captured in structured data alone).
- **Cluster 3 (male mild)**: Very small difference vs. placebo (+ 10.2 vs + 9.3) → No benefit; slow natural progression dominates

The Lanabecestat 50 mg arm does not rescue efficacy in any cluster: it shows either neutrality or slight-to-moderate worsening. The Lanabecestat 20 mg dose in Cluster 0-like patients remains the strongest candidate subgroup. The outcome illustrates our point: semantics-aware clustering, combined with dose stratification, could have identified that Lanabecestat 20 mg in females with mild-to-moderate baseline provided a promising signal, potentially leading to a smaller, targeted follow-up study rather than full termination.

For a better visualization of data, Summary of clusters for Placebo and Lanabecestat 50 mg is reported in Table 2.

**Table 2:** Clusters for the Lanabecestat 50 mg arm

Cluster	Description	Size	% Female	Mean Age	Placebo ADAS change	Placebo CDR-SB change	Lanabecestat 20 mg ADAS change	Lanabecestat 20 mg CDR-SB change	Lanabecestat 50 mg ADAS change	Lanabecestat 50 mg CDR-SB change	Interpretation – 50 mg Arm
0	Female-Dominant Mild-to-Moderate Progressors	~ 645	100%	72.4	+ 11.8	+ 3.22	** + 8.5** (benefit)	** + 2.62**(benefit)	+ 10.1	+ 3.05	**Modest/no clear benefit**Numerical trend worse than 20 mg, consistent with original trial futility
2	Hispanic Mixed-Sex Moderate Progressors	~ 119	46.2%	72.7	+ 7.3	+ 3.7	+ 12.7 (worse)	+ 3.8	+ 13.4	+ 4.1	**Worse than placebo and 20 mg**Possible harm signal in this subgroup
3	Male-Dominant Mild Progressors	~ 509	0%	71.3	+ 9.3	+ 3.19	+ 9.3 (neutral)	+ 3.60	+ 10.2	+ 3.85	**Slightly worse than placebo**No benefit; aligns with overall futility
1	Outlier/Data anomaly	1	—	—	—	—	—	—	—	—	Ignored

## Conclusions and future directions

We argue that, in principle, LLM-driven clustering can turn a failed trial into a partial success by uncovering semantically meaningful, explainable subgroups that have been traditionally overlooked. This approach may also help identify novel causal relationships between newly identified prognostic external factors and disease subtypes, disease onset, and disease evolution.

By capturing subtle semantic relationships, LLM-driven clustering can identify patient subgroups that are truly homogeneous in their treatment responses, leading to more precise efficacy assessments [7, 10, 13]. More accurate stratification reduces variability within study cohorts, potentially reducing required sample sizes and study durations, thereby speeding up drug development or enabling the use of already available drugs for highly stratified patient subsets [2]. Explanations generated by LLM agents, grounded in rich semantic and metadata context, provide clinicians and researchers with actionable insights, build trust, and help translate research findings into clinical practice [11].

The AI-driven patient stratification approach outlined in this position paper has significant potential to reduce costs in clinical studies by enabling early identification of target subgroups. Traditional trials often enroll broad cohorts (~ 2200 patients in AMARANTH) to account for heterogeneity, resulting in high costs for recruitment, screening and monitoring. By using LLM-enriched embeddings to cluster patients by semantic similarity (e.g., baseline cognitive scores, demographics and inferred disease phenotypes), the approach identifies responsive subgroups early—potentially during pilot phases or retrospective data analysis. Early subgroup identification also enables adaptive trial designs, in which non-responsive clusters (e.g., Clusters 2 or 3 in the simulation) are deprioritized mid-study, thereby avoiding prolonged futility. By surfacing hidden responders (e.g., slow progressors who benefit from 20 mg, but not 50 mg), the method prevents the investment of resources in ineffective broad trials. Retrospective application to existing data could repurpose “failed” datasets for targeted follow-ups, avoiding the need for entirely new studies. No trial is truly failed, only semantically unmined.

Also, the proposed approach shifts from “one-size-fits-all” to precision enrollment, potentially reducing per-trial costs while increasing success rates. However, it requires upfront investment in data infrastructure and validation to mitigate biases in LLM outputs. By leveraging LLMs and innovative, metadata-aware graph-embedding techniques, we can enable a new era of patient-centric clinical research, accelerate the discovery of effective therapies, and ultimately realize the promise of “precision” medicine. Moreover, identifying the specific, yet unidentified, variable factors that contribute to disease susceptibility, manifestations and personalized responses to treatment will help us devise strategies to either prevent disease onset or reduce its impact on both patients and society. Finally, by making a new effective therapy available to at least a subset of patients with a defined disease, this new approach may help maximize the return on drug development and reduce the burden on healthcare.

Our framework enables personalized therapies that are tailored to each patient’s unique biological and clinical profile. Further research is needed to integrate disparate data modalities (e.g., genomic, imaging, wearable device data and clinical notes) into a unified, metadata-aware embedding space, building upon the principles of LLM-enhanced graph embeddings [3] to develop systems that can perform real-time patient stratification, dynamically adjusting cohorts for adaptive trial designs, or providing immediate, personalized insights for point-of-care decision-making. With AI as the decoder, yesterday’s non-responders can become tomorrow’s precision cures.

The objectives we propose to the community for future research in this area include:

- *Integrating causal inference techniques with LLM-based stratification*: This integration promises not only to identify patient groups, but also to infer causal links between specific patient characteristics (including metadata attributes) and either treatment outcomes or specific factors in patients’ exposomes that affect disease onset, presentation and evolution.
- *Ethical Considerations and Bias Mitigation: *Addressing ethical concerns related to data privacy, algorithmic bias, and fairness in patient stratification, ensuring equitable access to personalized treatments and preventing the perpetuation of existing healthcare disparities. This will involve careful consideration of how metadata itself might introduce or mitigate bias.
- *Benchmarking and External Validation*: Rigorous benchmarking and external validation of the framework across diverse clinical domains, real-world patient populations and different healthcare systems are essential to demonstrate its generalizability, robustness, and clinical utility.
- *Interactive Explanation Interfaces*: Developing intuitive, interactive interfaces that allow clinicians to explore the patient clusters, query the LLM’s explanations and drill down into the underlying metadata that defines each group.

## Data Availability

In this MS, we used publicly available data from a previous clinical study to reanalyse it according to a new artificial-intelligence method allowing better patients’ stratification.
