# Correction to: Turning failure into success: how artificial intelligence can help personalize therapies and re-use patient data

**DOI:** 10.1007/s11302-026-10189-9

**Published:** 2026-09-17

**Authors:** Maria P. Abbracchio, Ernesto Damiani

**Affiliations:** Università Degli Studi Di Milano, Milan, Italy

**Correction to: Purinergic Signalling (2026) 22:54**

**https://doi.org/10.1007/s11302-026-10165-3**

The original version of the article unfortunately contained an error.

The correct data availability statement is shown below.

The analyses reported here were performed on a synthetic dataset (n = 1,273) whose variables correspond to those reported for the AMARANTH trial (NCT02245737). It contains no real patient data. The dataset was obtained by the authors in January 2026; we did not generate it, so its generative parameters are unknown. The file, as analyzed, is deposited at [https://doi.org/10.5281/zenodo.22072407], with a note that the final record is truncated. Registry metadata for NCT02245737 was downloaded from ClinicalTrials.gov.

